# Unveiling the influence of device stiffness in single macromolecule unfolding

**DOI:** 10.1038/s41598-019-41330-x

**Published:** 2019-03-21

**Authors:** G. Florio, G. Puglisi

**Affiliations:** 1 Dipartimento di Meccanica, Matematica e Management, Politecnico di Bari, Via Re David 200, I-70125 Bari, Italy; 2 Istituto Nazionale di Fisica Nucleare, Sezione di Bari, I-70126 Bari, Italy; 3 Dipartimento di Scienze dell’Ingegneria Civile e dell’Architettura, Politecnico di Bari, Via Re David 200, 70125 Bari, Italy

## Abstract

Single-molecule stretching experiments on DNA, RNA, and other biological macromolecules opened up the possibility of an impressive progress in many fields of life and medical sciences. The reliability of such experiments may be crucially limited by the possibility of determining the influence of the apparatus on the experimental outputs. Here we deduce a model that let us analytically evaluate such influence, fundamental for the interpretation of Single Molecule Force Spectroscopy experiments and intermolecular interactions phenomena. As we show, our model is coherent with previous numerical results and quantitively reproduce AFM experimental tests on titin macromolecules and P-selectin with variable probe stiffnesses.

## Introduction

The comprehension of the role of mechanical forces at the molecular level represents nowadays the focus of incredible efforts in many different research fields of Biology, Biomechanics, Material Engineering, Biomedical Sciences. Fundamental phenomena such as DNA and RNA hairpins unfolding and refolding in enzymatic activity, sensing of metabolites, transcription termination and attenuation, morphogenic phenomena, cell motility, focal adhesion and so on cannot be described without a comprehension of mechanical fields effect and macromolecular and cells force-response^1^.

In recent years the possibility of accurate experimental tests detecting protein and cells mechanical responses through Single Molecule Force Spectroscopy (SMFS) techniques has given a great impulse to understand the effects of mechanical forces in the natural environment. This scientific breakthrough has been achieved thanks to an increasing evolution of high-precision instruments such as Atomic Force Microscopes (AFM), optical tweezers, magnetic tweezers and microneedles.

Differently from temperature or chemical denaturant based classical manipulation techniques, SMFS allows for the choice of specific trajectories in complex *bumpy* energy manifolds, with the possibility of analyzing the relative stability of locally stable configurations and topological properties of the entire energy landscape^2–4^. An important example of this possibility is observed in the folding and refolding phenomena of the RNA or DNA secondary structure underlying gene transcription. Indeed, in this case SMFS experiments show that unzipping propagates through a single stranded/unstranded front (di-block behavior)^5^ whereas this is not observed for temperature of chemically induced denaturation. Moreover SMFS experiments are fundamental to analyze the mechanical response of the many proteins that are designated to withstand or transmit forces such as cytoskeletal or muscle protein macromolecules.

One of the main drawbacks of SMFS is that the observed force-end to end distance diagrams are strongly influenced by unavoidable effects due to the pulling device. Although a significant effort, both from a theoretical and experimental perspective, has been pursued on^6,7^, these effects are often neglected in many theoretical approaches and underestimated in the experimental field. In particular, the two main aspects to be analyzed in this perspective are rate of loading and probe stiffness effects. Regarding the former, we point out that, schematically, two different behaviors can be observed^8,9^. At high rate of loading, a rate dependent regime is observed and Kramer’s type relations are adopted with unfolding forces growing (logarithmically) with the loading rate^10,11^. More recently, in^12,13^ the authors compare analytical results with numerical simulations based on Brownian dynamics and show that it is possible to recover the unfolding rates taking into account corrections from the measuring apparatus.

At low rate of loading (*k*_*d*_*v* < 10 pN, where *k*_*d*_ is the device stiffness and *v* the pulling rate^8^), when the relaxation rate to the energy minimum of the molecular chain is much higher than the external loading rate, the force is rate-independent and it is influenced only by the stiffness *k*_*d*_. This rate-independent regime characterizes many SFMS experiments as well as many unfolding^14^ and detaching ligand-receptor phenomena in physiological regimes^8^ and is the focus of this paper.

It is important to remark that, as reported *e.g*. in^15^, different experimental devices can show force transducer stiffnesses differing of five order of magnitudes (from 10^−3^ to 100 N m^−1^). To the knowledge of the authors, the first theoretical analysis of the device stiffness effect on SMFS was delivered in^16^. There, based on a Statistical Mechanics approach, the behavior of a chain of elements with convex potential energy in series with an harmonic springs with variable stiffness reproducing the probe effect is studied. Numerical examples to describe the experimental behavior of poly (ethylene glycol) molecules have also been performed.

As we show in this paper, even more important is the effect of probe stiffness when one considers molecules constituted by elements exhibiting two (or more) ‘stable’ configurations. Two ideal extreme cases can be considered, depending on the ratio between the cantilever and the molecule stiffness^6,16^. In the case of stiff cantilevers (hard device) the system can be described using the Helmholtz ensemble: the molecule is held at a fixed extension with the corresponding force representing a ‘fluctuating’ dual variable. In the opposite case of soft cantilever (soft device), the Gibbs ensemble is adopted with an assigned force acting on the molecule and a fluctuating end-to-end length. The two ensembles can be shown to be equivalent in the thermodynamical limit with the Helmholtz and Gibbs free energies correlated by a Legendre transform^17^. As we show in this paper, completely different experimental responses can be observed for the same macromolecule under such different boundary conditions. For example in the fundamental case of protein macromolecules constituted by two-states (folded/unfolded) modules, the modification of the probe stiffness can induce a very different change in the response of the chain: the behavior can range from force-plateaux to sawtooth force-elongation diagrams corresponding to a cooperative and a non cooperative transition, respectively^17–19^. This results also in important variations of the force inducing the dissociation of a molecular complex^20^, the unfolding of a protein^21^, or of higher-order protein structure^22^.

In this perspective, in this paper we extend the analysis in^16^ by considering a chain of elements with non convex, two wells potential energies in series with an harmonic spring representing the device stiffness. To fix the ideas, we associate a different shape chain configuration to each energy well such as in the case of protein macromolecules^21^. In this case the chain is constituted by (*α*-helices or *β*-sheets) folded elements undergoing a conformational (unfolding) transition due to the elongation imposed by the pulling device. On the other hand, our approach can be extended to other fields where non convex (free) energies have been successfully adopted such as force induced martensitic phase transformation in shape-memory alloys^23^.

We recall that the possibility of deducing the hard and soft cases as limit models for a system with variable probe stiffness, was numerically shown in^16^ in the deduction of a model predicting the behavior of single polyethylene glycol chains AFM experiment. The analysis is more subtle when one considers local energy minimizers (metastable configurations) that can survive also in the thermodynamical limit^24^. The case of a chain with two-wells energy, in the limit of hard device, has been studied in^25^. In particular, in order to obtain an analytical expression of the partition function and analyze the thermodynamical limit, the authors use the approximation that we adopt here to extend the quadratic energy wells beyond the spinodal point. The transition to the soft regime has been studied using Monte Carlo numerical simulations in^26^. The importance of intermediate stiffness regimes is obvious: in real experiment one cannot expect a regime completely described by the hard or soft device limits. On the contrary, one would like to have a theory describing and predicting the behavior of the unfolding phenomenon in all intermediate regimes. In this paper we obtain explicit expressions for the partition function and force-strain relations that are valid in the *whole* range of values of the measuring device stiffness.

Interestingly, despite the simplicity of the proposed model, the comparison with AFM pulling behavior on titin macromolecule and P-selectin single module unfolding at different probe stiffnesses demonstrate its effectiveness in describing the influence of device stiffness in the experimental response of real biological molecules with particularly accurate predictivity.

It is important to remark the importance of analytical results regarding the mechanical interaction between an elastic link and a multidomain unfolding macromolecule. Indeed this is fundamental both in the perspective of bioinspired material design and in the important topic of evaluating the mechanical interaction between protein macromolecules and external elastic domains.

## Results

**Figure 1:**
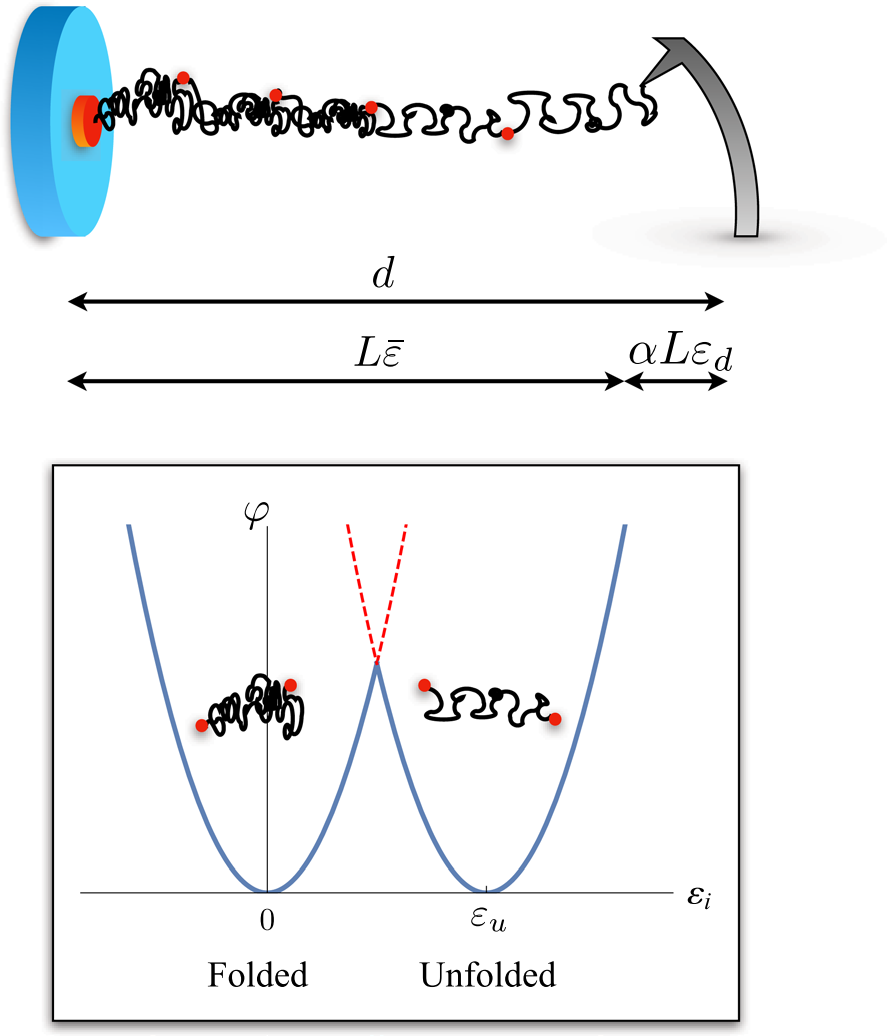
Scheme of an SFMS experiment for the analysis of the unfolding of a protein molecule constituted by modules undergoing a folded/unfolded transition here modeled through a two wells potential energy assumption.

Consider a SMFS experiment on a molecule constituted by bi-stable elements (see Fig. 1). We model the macromolecule as a chain of *N* masses connected by bistable springs and a probe acting on the *N*-th oscillator through a spring with stiffness *k*_*d*_.

The potential energy of the chain is ${V}_{c}={\sum }_{i=1}^{N}\,\frac{L}{N}\phi ({\varepsilon }_{i}),$ where *L* is the reference length of the molecule, *ε*_*i*_ is the strain of the *i*-th spring and *φ* its potential energy. Since the folded/unfolded transition of protein domains is typically an all or none transition, we assume for each domain a two wells energy $\phi ({\varepsilon }_{i})=\frac{{k}_{p}}{2}{({\varepsilon }_{i}-{\varepsilon }_{u}{\chi }_{i})}^{2}.$ Here *χ*_*i*_ is a ‘spin variable’ such that *χ*_*i*_ = 0 if the *i*-th spring is in the first (folded) state and *χ*_*i*_ = 1 if it is in the second (unfolded) state, with *ε*_*u*_ the reference strain of the second well. Observe that, to get analytical results, we approximate the energy wells by parabolic laws and that for simplicity of notation we assume that the spring stiffness *k*_*p*_ is the same in the two states. We also assume that the transition (Maxwell) force of the springs is zero. The following analysis is easily extended to the case when different stiffnesses in the two wells and non zero Maxwell force^18^ are considered.

The crucial aspect of our model is to consider the macromolecule and the measurement device as a single system  with a total potential energy

1 $$V={V}_{c}+{V}_{d}={\sum }_{i=1}^{N}\frac{L}{N}{k}_{p}\frac{{({\varepsilon }_{i}-{\varepsilon }_{u}{\chi }_{i})}^{2}}{2}+\alpha L{k}_{d}\frac{{\varepsilon }_{d}^{2}}{2}.$$

Here *V*_*d*_ is the energy of the measurement device, *ε*_*d*_ is the probe deformation and *αL* is its undeformed length. For small displacements (~150 nm) we can approximate the probe as a linear spring^27^. The potential energy clearly depends on the configuration assumed by the domains (folded or unfolded) described by the (internal) variables {*χ*_1_, .. *χ*_*N*_}. In the following, we sum over all configurations in order to obtain the canonical partition function of the system.

We model the SMFS experiment by assigning the total displacement *d* that is related to the strains by the following relation:

2 $$d={\sum }_{i=1}^{N}\frac{L}{N}{\varepsilon }_{i}+\alpha L{\varepsilon }_{d}=L(\bar{\varepsilon }+\alpha {\varepsilon }_{d}),$$

where we have introduced the average chain strain

3 $$\bar{\varepsilon }=\frac{1}{N}\,{\sum }_{i=1}^{N}{\varepsilon }_{i}.$$

As already mentioned, we analyze the fundamental effect of different values of *k*_*d*_ in the framework of equilibrium Statistical Mechanics^28^. In the following we first consider the zero-temperature case, especially interesting when rate-independent hysteresis is observed^18^, then we consider the important temperature effects.

### Zero temperature

The response of the considered system is regulated by its possibility to ‘explore’ the bumpy energy landscape. This, in turn, depends on three different time scales: the time scale of the external loading, the time scale of relaxation to the local minimizer and the time scale of exploring the whole energy landscape to relax to the global energy minimum. While, as we will show in the following section, temperature effects are crucial for the description of the system response (characterized by folded configurations where entropic effects play the major role), in this paragraph, following^19^, we consider a simplified analysis, neglecting entropic effects and considering two classical approaches based on the ability of the system to cross energy barriers. In the first case, the so-called *Maxwell convention*, the system is able to overcome all the energy barriers so that the configurations of the system always correspond to the global energy minima. On the other hand, the presence of local minima is fundamental to analyze the emergence of hysteresis cycles. In order to describe this possibility, we here consider the opposite hypothesis, *maximum delay convention*, where the system is not able to overcome any energy barrier, so that it stays in a local energy minimum until it becomes unstable and switches to the nearest energy well^19^.

#### Maxwell convention

Assume first that the configurations of the system are the global minima of the total potential energy. The equilibrium configurations are characterized by a constant force *F* with

4 $${\varepsilon }_{i}=\frac{F}{{k}_{p}}+{\varepsilon }_{u}{\chi }_{i}\,i=1,\ldots ,N,\,{\varepsilon }_{d}=\frac{F}{{k}_{d}}.$$

We then obtain the force-strain relation

5 $$F={k}_{p}(\bar{\varepsilon }-{\varepsilon }_{u}\bar{\chi }),$$

where $\bar{\chi }\,:\,=\frac{1}{N}\,{\sum }_{i=1}^{N}\,{\chi }_{i}\in [0,1]$ is the fraction of unfolded elements.

By using (2–5) we then obtain that at equilibrium

6 $$\bar{\varepsilon }=\gamma \delta +(1-\gamma ){\varepsilon }_{u}\bar{\chi },$$

where *δ* = *d*/*L* and

7 $$\gamma =\frac{{k}_{d}}{{k}_{d}+\alpha {k}_{p}}\in [0,1],$$

is the *main non dimensional parameter of the paper* measuring the relative stiffness of the probe *vs* the entire (chain plus probe) system. The ideal limits of ‘hard’ and ‘soft’ devices correspond to *γ* → 1 and *γ* → 0, respectively.

Using (1) and (5) it is possible to obtain the (non dimensionalized) force $\tilde{F}=\frac{F}{{k}_{p}}$ and potential energy $\tilde{V}=\frac{V}{{k}_{p}L}$ for the generic equilibrium configuration at given unfolded fraction $\bar{\chi }$:

8 $$\begin{array}{lllll}\tilde{F} & = & \tilde{F}(\delta ) & = & \gamma (\delta -{\varepsilon }_{u}\bar{\chi }),\\ \tilde{V} & = & \tilde{V}(\delta ) & = & \frac{\gamma }{2}{(\delta -{\varepsilon }_{u}\bar{\chi })}^{2}.\end{array}$$

Observe that all two-phase solutions with $\bar{\chi }\in ]0,1[$ are defined only for $|\tilde{F}|\le {\varepsilon }_{u}$. Thus, the existence domain of each two-phases equilibrium branch is

9 $$\delta \in [(\bar{\chi }-\frac{1}{\gamma }){\varepsilon }_{u},(\bar{\chi }+\frac{1}{\gamma }){\varepsilon }_{u}].$$

The fully folded state is defined for $\delta \le \frac{1}{\gamma }{\varepsilon }_{u}$ whereas the fully unfolded configuration for $\delta \ge (1-\frac{1}{\gamma }){\varepsilon }_{u}$.

One can deduce that all these solutions are metastable (local energy minima) due to the local convexity of the energy of both springs and probe. The global minima of the energy can be evaluated using (8). The two-phases configurations with phase fraction $\bar{\chi }$ corresponds to the global minimum for

10 $$\delta \in [(\bar{\chi }-\frac{1}{2N}){\varepsilon }_{u},\,(\bar{\chi }+\frac{1}{2N}){\varepsilon }_{u}].$$

The fully folded state represents the global minimum for $\delta \le \frac{1}{2N}{\varepsilon }_{u}$ whereas the fully unfolded configuration corresponds to the global minimum of the energy for $\delta \ge {\varepsilon }_{u}-\frac{1}{2N}{\varepsilon }_{u}$. We thus obtain the fundamental result at zero temperature: the unfolding force is constant and it depends on the relative stiffness of the probe with respect to the macromolecular chain. In particular, if we increase *δ* from the fully folded state then the chain starts to unfolds at the threshold

11 $${\tilde{F}}_{u}=\frac{\gamma }{2N}{\varepsilon }_{u}.$$

On the other hand, if we let the system refold by relaxing the probe and let *δ* decreases, the chain start to refold to go back to the primary folded state at the threshold

12 $${\tilde{F}}_{f}=-\,\frac{\gamma }{2N}{\varepsilon }_{u}.$$

Observe that these values should be considered as incremental force from the transition force of the bistable spring, here assumed null for simplicity of analytical expressions.

**Figure 2:**
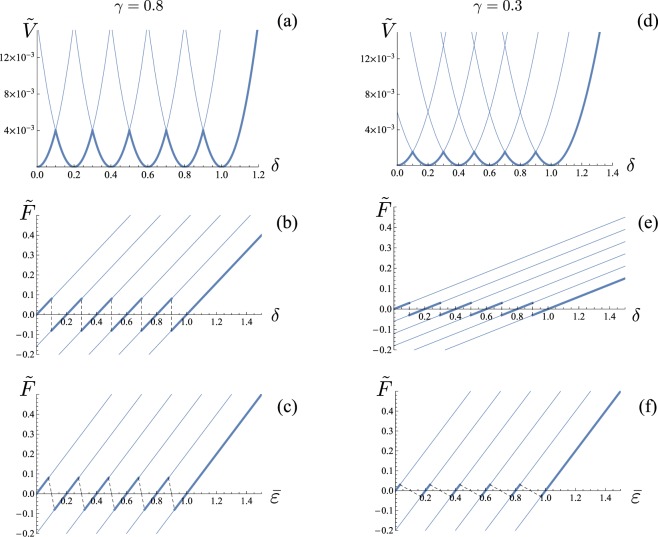
Plot of $\tilde{V}$ as a function of *δ* and of $\tilde{F}$ as a function of both *δ* and $\bar{\varepsilon }$ at zero temperature for a chain with *N* = 5 elements and *ε*_*u*_ = 1: *γ* = 0.8 (**a**–**c**) and *γ* = 0.3 (**d**–**f**).

In Fig. 2 we represent the important modifications of the obtained unfolding behavior depending on the relative stiffness parameter *γ*. Dashed lines represent the ‘jumps’ between different equilibrium branches under the Maxwell convention hypothesis that the system configurations correspond always to the global energy minima. Interestingly, as the device stiffness is decreased, not only the overall stiffness decreases (slope of the branches), but the force transition thresholds decrease in amplitude with an unavoidable influence of the pulling device stiffness.

We remark that in the considered model we neglect non local interactions. As a result, the configurations with the same fraction of unfolded domains are energetically independent on the number of folded/unfolded interfaces. The introduction of non local energy terms allows to determine the role of the loading device in determining also the number of interfaces of the energy minimum configurations^29^.

#### Maximum delay convention: hysteresis

Let us show how the hysteresis phenomenon emerges in our model and its dependence on the relative stiffness of the probe with respect to the chain measured by the parameter *γ* defined in (7). As a general remark, it is well known that, at constant rate, this non-equilibrium process is governed by several different time-scales^30^, related to the escape rates from local minima of the free energy and to the properties of energetic barriers (average time between escape attempts, average time between successive escapes and average lifetime of an individual energy minimum). On the other hand, in the following, we focus on the features of hysteresis in the rate-independent behavior which is dominant in the case of low temperature regimes^19,24^. Specifically, in the limit of zero temperature we assume, according with the so called maximum delay convention, that the system is not able to overcome any energy barrier.

**Figure 3:**
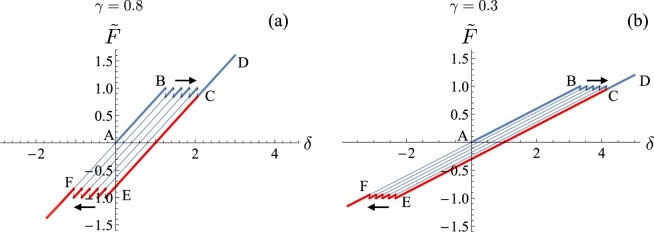
Plot of hysteresis cycles at zero temperature for a chain with *N* = 5 elements and *ε*_*u*_ = 1: *γ* = 0.8 (**a**) and *γ* = 0.3 (**b**). See the text for details about the sequence of loading-unloading path.

In Fig. 3 we plot two examples of hysteresis cycles for a chain with *N* = 5 in the cases of *γ* = 0.8 and *γ* = 0.3 (we refer to^18^ for all analytical details). If the system is loaded starting from the fully folded state, it follows the corresponding equilibrium path (AB in the figure) until the configuration becomes unstable and the system starts unfolding along a ‘sawtooth’ transition path (BC in the figure). If the displacement is further increased, the system follows elastically the fully folded branch (CD in the figure). If then the system is unloaded, it remains in this configuration until it becomes unstable (DE in the figure) and the reverse (unfolded/folded) transition is attained along another sawtooth plateau (EF in the figure). In this case, each jump from one branch to the other (dashed lines in the figure) occurs when the value of the total rescaled displacement *δ* reaches the bound of the existence domain defined in (9). This description corresponds to the so-called *maximum delay* strategy^19,24^. We notice that, starting from the origin, the effect of increasing *δ* strongly depends on *γ* and we obtain different cycles. The transition from the hard to the soft device regime (i.e., from large to small values of *γ*) corresponds to a reduction of the size of the jumps and to a decrease of the slope (see Fig. 2 for comparison).

### Non-zero temperature

In this section we analyze the fundamental temperature effect on the unfolding molecular behavior at different values of *γ* in the framework of equilibrium Statistical Mechanics^28^.

Following^25^, in order to get analytical result, we expand the two energy wells beyond the spinodal point where they intersect. In Fig. 1 we have marked these regions with the dashed branches of the parabola. As we discuss in Supplementary Material (SM), in the typical temperature range of living proteins this approximation is fully acceptable because the ‘artificial’ configurations with higher potential energy do not modify significantly the partition function.

Detailed calculations (see SM) deliver the canonical partition function *Z*_*N*_ of a chain of *N* elements and the measuring apparatus. We find

13 $${Z}_{N}={K}_{N}{(1-\gamma )}^{1/2}\sum _{p=0}^{N}\,(\begin{array}{c}N\\ p\end{array}){e}^{-\frac{\beta {k}_{p}l\gamma N}{2}{({\varepsilon }_{u}\frac{p}{N}-\delta )}^{2}},$$

where *K*_*N*_ is a constant, *β* = 1/*k*_*B*_*T*, *k*_*B*_ is the Boltzmann constant, *T* is the absolute temperature, *l* = *L*/*N* and the binomial coefficient gives the number of configurations of the chain with fraction $\bar{\chi }=\sum {\chi }_{i}/N=p/N$ of unfolded domains. Observe that the terms in the sum are Gaussian functions centered in *ε*_*u*_*p*/*N* and width proportional to $1/\sqrt{\beta {k}_{p}l\gamma N}$. At low temperature (large *β*) the Gaussian terms are ‘squeezed’ and contributions to *Z*_*N*_ are relevant only for values of *δ* very close to ${\varepsilon }_{u}\,p/N={\varepsilon }_{u}\bar{\chi }$ with *p* = 0, …, *N*.

The free energy is defined as ${{\boldsymbol{\Phi }}}_{N}=-\,\frac{1}{\beta }\,\mathrm{ln}\,{Z}_{N}$ and the expectation value of the force *F* applied to the system, conjugate to the displacement *δ*, is $\langle F\rangle =\frac{1}{L}\frac{\partial }{\partial \delta }{{\boldsymbol{\Phi }}}_{N}=-\,\frac{1}{\beta L}\frac{1}{{Z}_{N}}\frac{\partial }{\partial \delta }{Z}_{N}$. From Eq. (13) we thus find

14 $$\langle F\rangle ={k}_{p}\gamma (\delta -{\varepsilon }_{u}\langle \bar{\chi }\rangle ),$$

where

15 $$\langle \bar{\chi }\rangle =\frac{{\sum }_{p=0}^{N}(\begin{array}{c}N\\ p\end{array})\frac{p}{N}{e}^{-\frac{\beta {k}_{p}lN\gamma }{2}{({\varepsilon }_{u}\frac{p}{N}-\delta )}^{2}}}{{\sum }_{p=0}^{N}(\begin{array}{c}N\\ p\end{array}){e}^{-\frac{\beta {k}_{p}lN\gamma }{2}{({\varepsilon }_{u}\frac{p}{N}-\delta )}^{2}}}.$$

Here $\langle \bar{\chi }\rangle$ is the expectation value of the fraction $\bar{\chi }=p/N$ of unfolded domains. Similarly (see SM), the expectation value of the average strain in Eq. (3) is

16 $$\langle \bar{\varepsilon }\rangle ={\varepsilon }_{u}\langle \bar{\chi }\rangle +\gamma (\delta -{\varepsilon }_{u}\langle \bar{\chi }\rangle ).$$

Finally, from Eqs (14) and (16) we obtain the *central* result of the paper giving the force-strain relation at assigned temperature and relative stiffness *γ*

17 $$\langle F\rangle ={k}_{p}(\langle \bar{\varepsilon }\rangle -{\varepsilon }_{u}\langle \bar{\chi }\rangle )$$

as implicit functions of the total displacement *δ*. This equation fully characterizes the material response of the macromolecule for the whole range of stiffness ratio *γ*.

**Figure 4:**
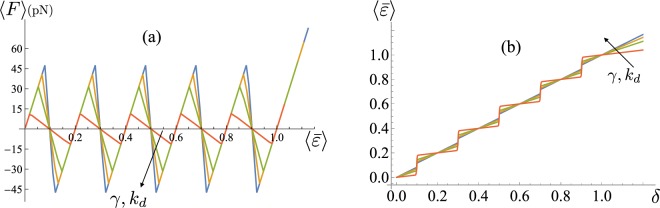
(**a**) Force-strain curves for different values of *γ* (and *k*_*d*_). Here *N* = 5, *ε*_*u*_ = 1, *T* = 300 K, *l* = 30 nm and ${\bar{k}}_{\exp }={k}_{p}/Nl=4\,\text{pN}/\text{nm}$. The curves are obtained for ${\bar{k}}_{d}={k}_{d}/(\alpha L)=1,5,10,20\,\mathrm{pN}/\mathrm{nm}$ corresponding to $\gamma \simeq 0.2,0.56,0.71,0.83$, respectively (see SM). (**b**) $\langle \bar{\varepsilon }\rangle$–*δ* curves for the same parameters of (**a**).

In Fig. 4a we plot a set of force-strain curves obtained for different values of *γ* corresponding to different stiffnesses of the probe *k*_*d*_. As described previously, different values of the device stiffness alter quantitatively and qualitatively the macromolecule response. Thus the unfolding force decreases as *k*_*d*_ decreases with the transition becoming cooperative. Observe that, as shown in the Fig. 4b, for decreasing *k*_*d*_ (i.e. softer devices) each unfolding transition corresponds to localized strain jumps. In the opposite case of hard devices the strain evolution is smoother.

**Figure 5:**
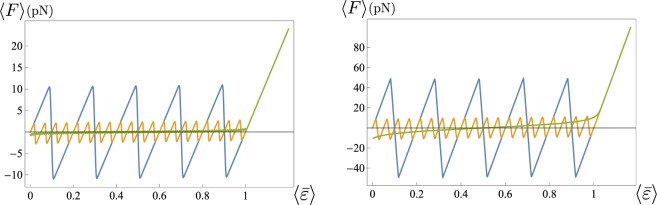
Force-strain curve with variable number of elements *N* = 5,20,100. Left: fixed element length *l* = 30 nm and ${\bar{k}}_{p}={k}_{p}/l=4\,\mathrm{pN}/\mathrm{nm}$. Right: fixed total length *L* = *Nl* = 150 nm and ${\bar{k}}_{\exp }={k}_{p}/Nl=4\,\text{pN}/\text{nm}$. Larger values of *N* correspond to smaller amplitude of the oscillations. Here *ε*_*u*_ = 1, *T* = 300 *K* and ${\bar{k}}_{d}={k}_{d}/(\alpha L)=20\,\mathrm{pN}/\mathrm{nm}$.

In Fig. 5 we study the interesting effect of increasing *N*. As expected, the amplitude of the oscillations decreases as *N* grows. We notice that in one case (left figure) we have increased *N* keeping *l* fixed. On the other hand, in Fig. 5 (right) we have increased *N* keeping *L* = *Nl* fixed. As the figure shows, the effect of stiffness variation is particularly important for small systems whereas it becomes negligible in the thermodynamical system analyzed in the following paragraph.

**Figure 6:**
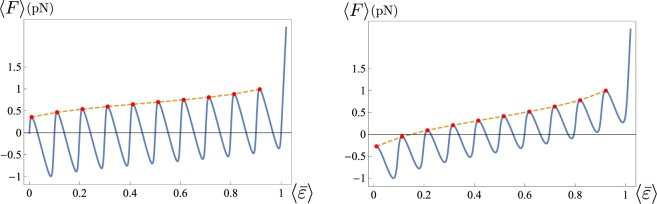
Force-strain curve showing the hardening effect of local maxima of *F* increasing with $\langle \bar{\varepsilon }\rangle$. Here *N* = 10, *ε*_*u*_ = 1, *T* = 300 K (left), *T* = 600 K (right), *l* = 30 nm and ${\bar{k}}_{\exp }={k}_{p}/Nl=0.4\,\mathrm{pN}/\mathrm{nm},{\bar{k}}_{d}={k}_{d}/(\alpha L)=0.1\,\mathrm{pN}/\mathrm{nm}$ (giving *γ* = 0.2).

Finally, observe that, in agreement with the AFM unfolding experiments on biological macromolecules such as titin^21^, the values of the local maxima of *F* (corresponding in the experiment to the forces for the successive unfolding of *β*-sheets domains) are not constant, but increase with $\langle \bar{\varepsilon }\rangle$ (*hardening effect*, see Fig. 6). This effect can be interpreted observing that as $\langle \bar{\varepsilon }\rangle$ grows the number *p* of unfolded domains is increased. Correspondingly the number of available configurations with *p* + 1 unfolded domains decreases. Interestingly, our tests show that the hardening effect is more evident for higher temperatures (see Fig. 4) and larger number *N* of domains (see Fig. 5). In particular, in the limit of soft device this effect leads to a transition from a non cooperative to a cooperative unfolding behavior. While in^31^ this effect has been addressed to a possible inhomogeneity of the unfolding domains, leading to different unfolding energies, it is important to remark that according with our results the experimentally observed hardening effect can be described as a purely temperature effect.

### Thermodynamical limit

Many protein materials are constituted by macromolecules with a large number of *β*-sheets or *α* helices folded domains undergoing conformational transformation under increasing end-to-end distance. Therefore, it is interesting to consider the limit of growing number of domains *N* → +∞.

We begin by searching for the asymptotic expression of the function $\langle \bar{\chi }\rangle$ in Eq. (15). Using the saddle point method (see SM for details) we find the expectation value of the unfolded fraction

18 $$\langle \bar{\chi }\rangle  \sim {\chi }_{c}(\delta ),$$

where *χ*_*c*_ ∈ (0, 1) is the minimum of the function

19 $$f(x)=S(x)+\tilde{\beta }{({\varepsilon }_{u}x-\delta )}^{2}.$$

Here

20 $$S(x)=x\,\mathrm{ln}\,x+(1-x)\,\mathrm{ln}\,(1-x)$$

and $\tilde{\beta }=\beta l{k}_{p}\gamma /2$. Finally, we have

21 $$\langle F\rangle  \sim {k}_{p}\gamma (\delta -{\varepsilon }_{u}{\chi }_{c})$$

and

22 $$\langle \bar{\varepsilon }\rangle  \sim {\varepsilon }_{u}{\chi }_{c}+\gamma (\delta -{\varepsilon }_{u}{\chi }_{c}).$$

**Figure 7:**
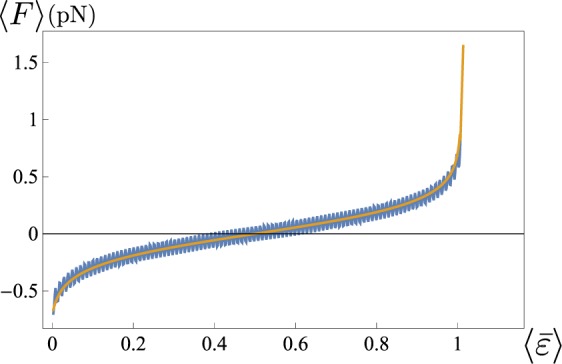
Comparison of the force-strain curves obtained in the thermodynamical limit (monotonic curve) and for *N* = 100 (chainsaw curve). Here *ε*_*u*_ = 1, *T* = 300 K, *l* = 30 nm, ${\bar{k}}_{p}={k}_{p}/l=4\,\mathrm{pN}/\mathrm{nm},\gamma =0.7$.

In Fig. 7 we show the agreement of the force-strain curves obtained in previous section for *N* = 100 and the thermodynamical limit case in Eqs (21 and 22).

#### Comparison with experimental results

**Figure 8:**
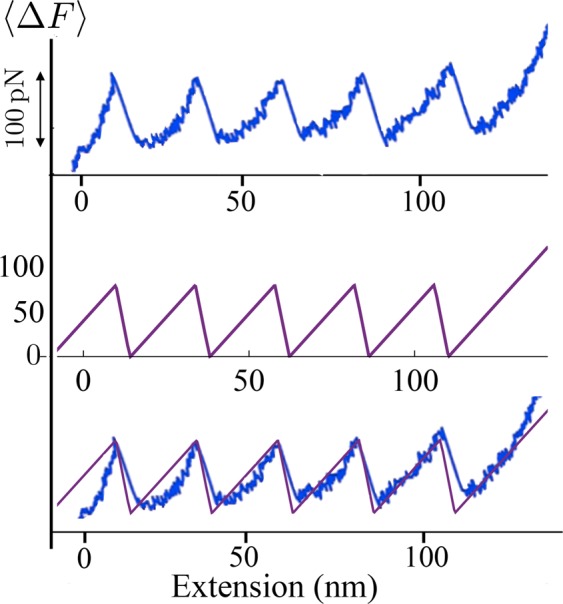
Comparison of the the force-strain curves obtained from the model with the experimental results (see^32^). We have fixed *N* = 5, *ε*_*u*_ = 1, *T* = 300 K, *l* = 24 nm and ${\bar{k}}_{\exp }={k}_{p}/Nl=4\,\mathrm{pN}/\mathrm{nm},{\bar{k}}_{d}={k}_{d}/(\alpha L)=20\,\mathrm{pN}/\mathrm{nm}$ (corresponding to $\gamma \simeq 0.86$).

In order to show the predictive properties of the proposed model, in Fig. 8 we compare the theoretical response with the AFM experimental unfolding behavior of titin reported in^32^. In the theoretical curve we assign the spring constant of the AFM used in those experiment ($\simeq \,20\,\mathrm{pN}/\mathrm{nm}$) and we set the stiffness of the titin as ${\bar{k}}_{\exp }=4\,\mathrm{pN}/\mathrm{nm}$ which, as we show in SM, is consistent with typical values of titin PEVK unfolding energies (see^31,33^). In the figure we have indicated 〈Δ*F*〉 because in our model the Maxwell stress of the two-wells potential energy is set to zero, but the average unfolding force of the proposed model can be easily calibrated on the experimental results by simply considering a non-zero Maxwell stress.

**Figure 9:**
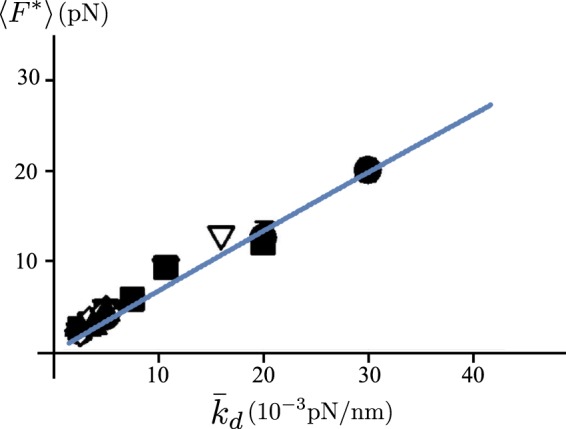
Dependence of the rupture force on the spring constant of a (very soft) transducer compared with experimental results (see^34^). We have fixed *N* = 1, *ε*_*u*_ = 35, *T* = 300 K, *l* = 40 nm and ${\bar{k}}_{p}=1\,\mathrm{pN}/\mathrm{nm},{\bar{k}}_{d}={k}_{d}/(\alpha L)$ varying from 1 × 10^−3^ pN/nm to 40 × 10^−3^ pN/nm. Different symbols correspond to different loading rates.

To further test our results against the experimental behavior, in Fig. 9 we reproduce the dependence of the rupture force 〈F^*^〉 for dissociating P-selectin-PSGL-1 bond on the stiffness of the transducer, experimentally determined in^34^. To this scope, following^35^, we describe the protein rupture as a transformation of a system with a two-wells potential energy and second minimum ‘far’ from the first one, *i.e*. ${\varepsilon }_{u}\gg 1$. Thus, we identify the fracture force 〈F^*^〉 with the maximum attained value of 〈*F*〉 as the rescaled total displacement *δ* is increased. The values of the parameters are taken from^34^ except *ε*_*u*_ (fixing the energy barrier) whose value is chosen in order to fit the experimental results. As the figure shows we obtain a linear trend quantitatively reproducing the experimental behavior.

#### Comparison with the Wormlike Chain model

**Figure 10:**
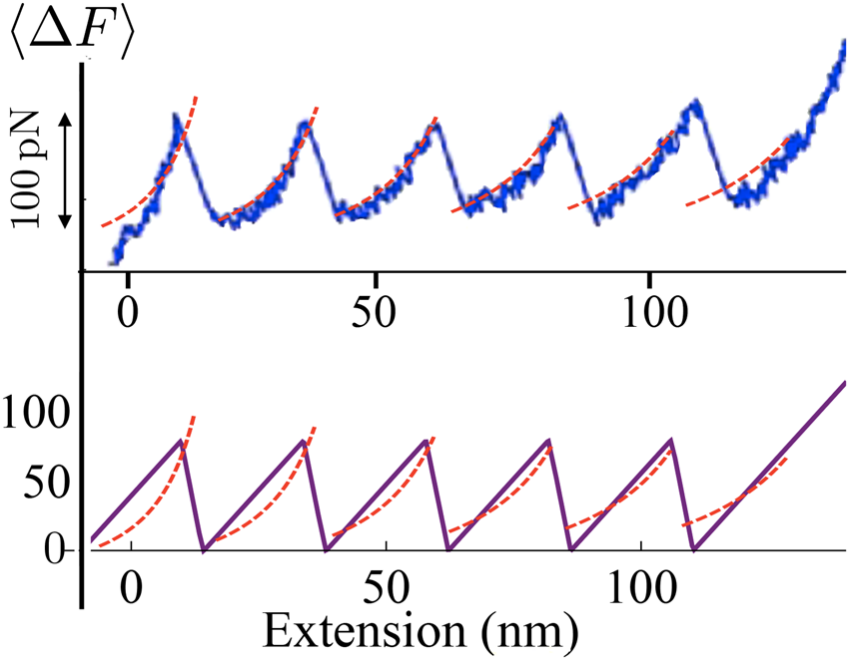
Comparison of the the force-strain curves obtained the wlc model (dashed lines) with experimental results (top, see^32^ and Fig. 8) and the results form our model (bottom, same parameters of Fig. 8). The parameters of the WLC are consistent with those reported in^32^: persistence length *l*_*p*_ = 0.4 nm, increase of the contour length *l*_*c*_ = 28 nm and device stiffness ${\bar{k}}_{d}\mathrm{=20}\,\mathrm{pN}/\mathrm{nm}$.

We remark that in order to keep analytical simplicity we have assumed a parabolic behavior of the energy for folded and unfolded configurations of the domains. While the experimental comparison in previous subsection confirmed the ability of determining the influence of the external loading device on the unfolding thresholds, we may observe that our energetic assumption leads to a linear global force displacement diagrams. In Fig. 10 we show the different behavior that is obtained when we consider the Wormlike Chain energy widely used to reproduce the behavior of biological macromolecules^36^. In this case, following the approach proposed in^31^, we neglect the entropic energy terms as in the zero-temperature approximation considered in Fig. 2 and we neglect the deformability of the folded domains. We refer to the Supplementary Material and^31^ for analytical details. Notwithstanding the fact that one observes a deviation from the linear behavior of the force obtained from our model, the predictions about the values of the measured force and the location of the jumps are consistent. On the other hand, we remark that, differently from the theoretical two wells model and the experimental behavior, in this case the unfolding force decreases with the unfolded fraction. This is due to the neglect of the temperature effects. Moreover, the adoption of the WLC energy does not let us obtain analytical formulations regarding the partition function that is the main aim of this paper.

## Discussion

The comprehension of the response of macromolecules is fundamental in many fields of biology, medicine and bio-inspired materials engineering. Indeed, the incredible elasticity and recovery properties of bio-materials result from the features of the constituent macromolecules. In this paper, we deduced a simple, analytical and effective description of the macromolecule behavior modeled as a chain of elements undergoing conformational transitions (such as in *β*-sheets and *α*-helices unfolding). SFMS techniques constituted in the last decades the main experimental tools to determine the protein macromolecules folding and refolding behavior and the mechanical behavior of biomolecules and cells. As we have shown in this paper, the only way to interpret such experiments is to model the macromolecule and the measuring device as a unique system (both at zero, see SM, and non-zero temperature). Here, for the first time, we deliver explicit analytic solutions describing the force *vs* end-to-end distance diagram as a function of a main non-dimensional parameter measuring the relative device versus macromolecule’s stiffness. As this parameter is varied, the model describes the experimentally observed regimes going from sawtooth transitions in the case of isometric (hard device ≡ *k*_*d*_ → ∞) conditions to the cooperative type (force plateau) transition observed for the assigned force experiments (soft device ≡ *k*_*d*_ → 0). Such crucial effect is often ignored in the interpretation of the experimental results^26^. We believe that the approach here proposed and its possible extensions can represent an important step forward in the field. In particular, the presence of mechanical interactions for proteins, cells and biological tissues play a crucial role in many fundamental diseases and biological functions^37^. In this perspective, we observe that a direct extension of our approach to such problems can give a new theoretical framework correctly considering the boundary conditions, *e.g*. for biomaterial growth and medical pathologies.

## Supplementary information

Supplementary Material
