# Supplementary material for: Unveiling the influence of device stiffness in single macromolecule unfolding
Source: Sci Rep. 2019 Mar 21;9:4997. doi: 10.1038/s41598-019-41330-x (PMC6428835; doi:10.1038/s41598-019-41330-x)
Supplement: Supplementary file 1 — Supplementary Material [file 41598_2019_41330_MOESM1_ESM.pdf]

Supplementary Material
“Unveiling the influence of device stiffness in single macromolecule unfolding”

G. Florio, G. Puglisi

In the following the formulas of the supplementary materials are designed by SM whereas the equations of the main paper are recalled without SM.

Basic definitions and energetic considerations

The Hamiltonian of the system can be written as

$$H = E_k + V, \tag{SM1}$$

where E_k and V take into account the contributions of kinetic and potential energies, respectively. In particular, we have

$$E_k = \sum_{j=1}^{N} \frac{N p_i^2}{2m} + \frac{p_{N+1}^2}{2M} \tag{SM2}$$

where p_j, $j = 1, \ldots N+1$ are the linear momenta, m/N and M denote the mass of each element of the chain and the representative mass of the apparatus, respectively. On the other hand, V contains both the non-convex potential energies of the bistable elements and the elastic contribution describing the interaction between the N-th element of the chain and the measuring apparatus.

Before reconnecting to the expressions adopted in the main paper in terms of strains, it is useful for the following calculations to introduce the variables $\xi_i = x_i - x_{i-1}$ representing the displacement difference between the elements i and $i-1$, with the convention ξ_0=0 and $\xi_{N+1} = \xi_d$ [see also Eq.(1)]. We have

$$V = \sum_{j=1}^{N} \Phi(\xi_i) + \frac{\bar{k}_d}{2} (\xi_{N+1})^2 \tag{SM3}$$

and

$$\Phi(\xi_j) = \frac{\bar{k}_p}{2} (\xi_j - a_j)^2. \tag{SM4}$$

The material constants $\bar{k}_p$ and $\bar{k}_d$ represent the stiffness of the bistable elements in the chain and of the measuring device, respectively. They are related to the constant in Eq. 1 of the main paper by the following relations:

$$\bar{k}_p = k_p/(L/N) = k_p/l, \quad \bar{k}_d = k_d/(\alpha L). \tag{SM5}$$

Notice that a_j can assume two different (non negative) values corresponding to the two different (folded and unfolded) minima $a_j^{(1)}, a_j^{(2)}$. As a preliminary consideration, we notice that in the following we introduce a simplification, i.e. we expand the two energy wells in Eq.(SM4) on the whole real line, beyond the spinodal point where they intersect. In Fig. 1 of the main paper we have marked these regions with the dashed branches of the parabola. This simplifies the calculations and let us obtain analytical expressions.

The partition function in the canonical ensemble takes the form

$$Z_N = \sum_{\{a_1,\dots,a_N\}\in\{a^{(1)},a^{(2)}\}^N} \int_{\mathbf{R}^{2(N+1)}} dp_1 \dots dp_{N+1} d\xi_1 \dots d\xi_{N+1} e^{-\beta H} \delta\left(\sum_{i=1}^{N} \xi_i + \xi_{N+1} - d\right), \tag{SM6}$$

where $\beta = 1/k_B T$, k_B is the Boltzmann constant, T the absolute temperature and we have used the Dirac delta in order to include the constraint

$$d = \sum_{i=1}^{N} \xi_i + \xi_{N+1} \tag{SM7}$$

fixing the total length d. We notice that, due to the structure of the Hamiltonian, the integrals over the momenta are Gaussian and reduce to the constant

$$A_N = (2\pi)^{(N+1)/2} \left(\frac{m}{N\beta}\right)^{N/2} \left(\frac{M}{\beta}\right)^{1/2}. \tag{SM8}$$

Thus, the partition function takes the form

$$Z_N = A_N \sum_{\{a_1,\dots,a_N\}\in\{a^{(1)},a^{(2)}\}^N} \int_{\mathbf{R}^{N+1}} d\xi_1 \dots d\xi_{N+1} e^{-\beta V} \delta\left(\sum_{i=1}^{N} \xi_i + \xi_{N+1} - d\right). \tag{SM9}$$

The constraint can be used to perform the integral over ξ_{N+1} so that

$$Z_N = A_N \sum_{\{a_1,\dots,a_N\}\in\{a^{(1)},a^{(2)}\}^N} \int_{\mathbf{R}^{N}} d\xi_1 \dots d\xi_N e^{-\beta\left(\sum_{j=1}^N \Phi(\xi_j) + \frac{\bar{k}_d}{2}\left(\sum_{i=1}^N \xi_i - d\right)^2\right)}. \tag{SM10}$$

Finally, we can rewrite the problem in terms of the strains ε_i using the transformation

$$\xi_i = \frac{L}{N}\varepsilon_i, \quad i = 1,\dots N. \tag{SM11}$$

We obtain

$$Z_N = C_N \sum_{\{\chi_1,\dots,\chi_N\}\in\{0,1\}^N} \int_{\mathbf{R}^{N}} d\varepsilon_1 \dots d\varepsilon_N e^{-\beta \frac{lk_p}{2}\left(\sum_i (\varepsilon_i - \varepsilon_u \chi_i)^2 + \frac{N\gamma}{1-\gamma}\left(\delta - \frac{1}{N}\sum_i \varepsilon_i\right)^2\right)}, \tag{SM12}$$

where $C_N = A_N (L/N)^N$, $\varepsilon_u \chi_i = a_i/(L/N)$, $l = L/N$, $\delta = d/L$. Notice that as in the case of zero temperature we have used the main dimensionless parameter γ defined in Eq.(7) of the main paper. We have that

$$\gamma = \frac{k_d}{k_d + \alpha k_p} = \frac{\bar{k}_d}{\bar{k}_d + \bar{k}_p/N}. \tag{SM13}$$

We notice that the stiffness of the macromolecule $\bar{k}_{exp}$ measured during an experiment corresponds to the stiffness of N springs in series. Therefore, we have

$$\bar{k}_{exp} = \bar{k}_p/N = k_p/Nl. \tag{SM14}$$

Thus, we find

$$\gamma = \frac{\bar{k}_d}{\bar{k}_d + \bar{k}_p/N} = \frac{\bar{k}_d}{\bar{k}_d + \bar{k}_{exp}}. \tag{SM15}$$

In the following we will set, without loss of generality, the two minima of the potential wells as $\chi_i = 0$ and $\chi_i = 1 > 0, i = 1, \ldots, N$.

A remark is now in order. Based on these definitions and corresponding energetic considerations, we may justify the choice of $\bar{k}_{exp} = 4$ pN/nm and $l = 24$ nm adopted in the reproduction of Fig. 8 of the main paper. Indeed, in order to get analytical results, we assumed a parabolic behavior in each well instead *e.g.* of a more realistic Wormlike Chain law. As a result, with these parameters the dissipation associated to each jump is

$$Q = \frac{1}{2}\bar{k}_{exp}\, l^2 = 1152\,\text{pN nm} \simeq 280\, k_B T \tag{SM16}$$

at 300 K. This value is in agreement with the values estimated in[1] based on the experiments performed on PEVK domains[2].

Partition function

A simple Gaussian integration of Eq.(SM12) gives

$$Z_N = C_N \left(\frac{2\pi}{\beta k_p l}\right)^{N/2} (1-\gamma)^{1/2} \sum_{\{\chi_1,\ldots,\chi_N\}\in\{0,1\}^N} e^{\frac{\beta k_p l}{2}\left(\sum_i (\varepsilon_u \chi_i + \frac{\gamma}{1-\gamma}\delta)^2 - \frac{\gamma}{N}(\sum_i(\varepsilon_u\chi_i + \frac{\gamma}{1-\gamma}\delta))^2 - \sum_i \varepsilon_u^2 \chi_i^2 - N\frac{\gamma}{1-\gamma}\delta^2\right)}. \tag{SM17}$$

The summation over the configurations $\{\chi_1, \ldots, \chi_N\}$ can be rephrased in terms of the fraction p/N of unfolded domains. In particular, we have

$$\bar{\chi} = \frac{\sum_i \chi_i}{N} = \frac{\sum_i \chi_i^2}{N} = p/N. \tag{SM18}$$

Using this result, we can find the final form of the partition function describing the chain *and* the measuring apparatus:

$$Z_N = K_N (1-\gamma)^{1/2} \sum_{p=0}^{N} \binom{N}{p} e^{-\frac{\beta k_p l \gamma N}{2}(\varepsilon_u \frac{p}{N} - \delta)^2}, \tag{SM19}$$

where

$$K_N = C_N \left(\frac{2\pi}{\beta k_p l}\right)^{N/2}, \tag{SM20}$$

and we have used the binomial coefficient in order to count the number of configurations of the chain with fraction p/N of unfolded domains.

The expectation value of the average strain $\bar{\varepsilon}$ (denoted as $\langle\bar{\varepsilon}\rangle$) can be evaluated as follows. We can use the partition function and the definition of average of a quantity in the canonical ensemble:

$$\langle\bar{\varepsilon}\rangle = \frac{1}{Z_N} C_N \sum_{\{\chi_1,\ldots,\chi_N\}\in\{0,1\}^N} \int_{\mathbf{R}^N} d\varepsilon_1 \ldots d\varepsilon_N \left(\frac{1}{N}\sum_i \varepsilon_i\right) e^{-\frac{\beta k_p l}{2}\left[\sum_i (\varepsilon_i - \varepsilon_u \chi_i)^2 + \frac{N\gamma}{1-\gamma}(\delta - \frac{1}{N}\sum_i \varepsilon_i)^2\right]}. \tag{SM21}$$

It is straightforward to show that

$$\frac{1}{L Z_N}\frac{\partial}{\partial \delta} Z_N = -\beta k_p \frac{\gamma}{1-\gamma}(\delta - \langle\bar{\varepsilon}\rangle)\,. \tag{SM22}$$

We thus find

$$\langle \bar{\varepsilon} \rangle = \delta - \frac{1-\gamma}{k_p\gamma}\left(-\frac{1}{\beta}\frac{1}{LZ_N}\frac{\partial}{\partial\delta}Z_N\right) = \delta - (1-\gamma)(\delta - \varepsilon_u\langle\bar{\chi}\rangle) = \varepsilon_u\langle\bar{\chi}\rangle + \gamma(\delta - \varepsilon_u\langle\bar{\chi}\rangle), \tag{SM23}$$

where

$$\langle\bar{\chi}\rangle = \frac{\sum_{p=0}^{N}\begin{pmatrix} N \\ p \end{pmatrix}\frac{p}{N}e^{-\frac{\beta k_p l N\gamma}{2}(\varepsilon_u\frac{p}{N}-\delta)^2}}{\sum_{p=0}^{N}\begin{pmatrix} N \\ p \end{pmatrix}e^{-\frac{\beta k_p l N\gamma}{2}(\varepsilon_u\frac{p}{N}-\delta)^2}} \tag{SM24}$$

is the expectation value of the fraction of unfolded domains. From these results we obtain (15-17) of the main paper.

Thermodynamical limit

Let us start considering the sum

$$g(\delta) = \sum_{p=0}^{N}\begin{pmatrix} N \\ p \end{pmatrix}e^{-\frac{\beta k_p l\gamma N}{2}(\varepsilon_u\frac{p}{N}-\delta)^2} \tag{SM25}$$

which appears in the expression of the partition function (SM17). We can introduce the variable $x = p/N$. In the limit of large N, by using the Stirling approximation $n! \simeq (n/e)^n\sqrt{2\pi n}$ for $n \gg 1$, we have

$$g(\delta) \simeq \sqrt{\frac{N}{2\pi}}\int_0^1 dx\sqrt{\frac{1}{x(1-x)}}e^{-N\left[S(x)+\tilde{\beta}(\varepsilon_u x-\delta)^2\right]}, \tag{SM26}$$

where $\tilde{\beta} = \beta l k_p\gamma/2$ and we have defined the entropy

$$S(x) = x\ln x + (1-x)\ln(1-x). \tag{SM27}$$

In the limit $N \to +\infty$ we can use the saddle point method and reduce Eq.(SM26) to a Gaussian integral. The explicit calculation gives

$$g(\delta) \sim \frac{1}{\sqrt{1+2\tilde{\beta}\varepsilon_u\chi_c(1-\chi_c)}}e^{-N\left[S(\chi_c)+\tilde{\beta}(\varepsilon_u\chi_c-\delta)^2\right]}, \tag{SM28}$$

where $\chi_c \in (0,1)$ is the minimum of the function

$$f(x) = S(x) + \tilde{\beta}(\varepsilon_u x - \delta)^2. \tag{SM29}$$

An analogous calculation shows that

$$\begin{aligned} h(\delta) &= \sum_{p=0}^{N}\begin{pmatrix} N \\ p \end{pmatrix}\frac{p}{N}e^{-\frac{\beta k_p l\gamma N}{2}(\varepsilon_u\frac{p}{N}-\delta)^2} \\ &\sim \frac{\chi_c}{\sqrt{1+2\tilde{\beta}\varepsilon_u\chi_c(1-\chi_c)}}e^{-N\left[S(\chi_c)+\tilde{\beta}(\varepsilon_u\chi_c-\delta)^2\right]}. \end{aligned} \tag{SM30}$$

We thus have

$$\langle\bar{\chi}\rangle = \frac{h(\delta)}{g(\delta)} \sim \chi_c(\delta), \tag{SM31}$$

and, finally, Eq.(21)-(22) of the paper.

Numerical test of the energy approximation

In this section we include some numerical results in order to confirm the validity of our approximations (extension of the parabolic function beyond the spinodal point) in the chosen range of temperature and values for the parameters of the system. In particular, in Fig. 1 we compare the force strain curves obtained via Eq.(14) of the main paper and the numerical integration of the partition function without approximation. Observe that, even in the case of very high temperature (T = 3000K), the approximation is very robust and leads to a result perfectly consistent with the numerical counterpart. Moreover, we test the approximation for different values of the spring constant of the measurement apparatus. The results are shown in Fig. 2 supporting the validity of the approximation considered in the paper.

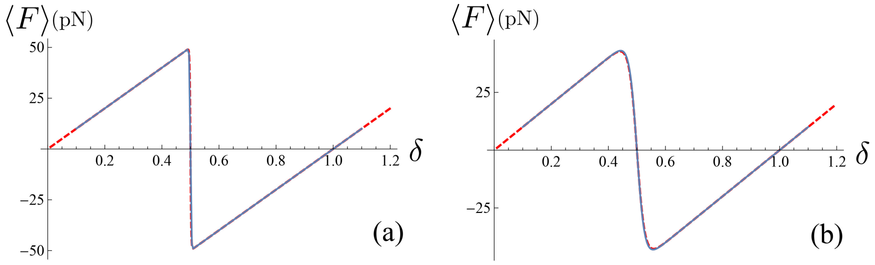

Figure 1. Comparison of the force-strain curves obtained using the analytical formula (14), dashed line, and the numerical integration of the partition function without the approximation beyond the spinodal point described in the text. We have considered two differerent temperatures:(a) T = 300 K, (b) T = 3000 K. In both cases we have fixed $N = 1$ with $l = 30\,\text{nm}, \varepsilon_u = 1, \bar{k}_d = k_d/(\alpha L) = 20\,\text{pN/nm}$ and $\bar{k}_p = k_p/l = 4\,\text{pN/nm}$.

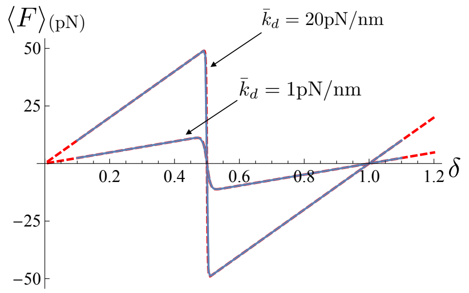

Figure 2. Comparison of the force-strain curves obtained using the analytical formula (14), dashed line, and the numerical integration of the partition function without the approximation beyond the spinodal point described in the text. We have considered two different spring constant of the measurement apparatus. In both cases we have fixed $N = 1$ with $T = 300\,\text{K}, l = 30\,\text{nm}, \varepsilon_u = 1$ and $\bar{k}_p = k_p/l = 4\,\text{pN/nm}$.

Wormlike chain energy

To reproduce the nonlinearity of the force-displacement equilibrium branches, following[1] we neglect the elasticity and the elastic energy of the folded domains, whereas we consider a WLC energy for the unfolded domains of the type

$$V_c^{(i)} = V_c(\eta_i, \chi_i) = \chi_i l_c \frac{k_B T}{l_p} \frac{\eta_i^2}{1-\eta_i} + Q, \qquad i = 1, \dots N \tag{SM32}$$

where l_p is the persistence length, l_c is the contour length of the single domain, k_B is the Boltzmann constant, T the absolute temperature, and Q is the hentalpic contribution representing the energy expended

to unfold the domain. Here $\eta_i = d_i/l_c$ is the relative deformation of the i-th domain, where d_i is its deformed length. The total energy, analogous to Eq.(1) of the main paper, is then

$$\Phi_{\text{tot}} = \sum_i V_c^{(i)}(\eta_i, \chi_i) + \bar{k}_d \frac{l_d^2}{2}, \tag{SM33}$$

where we have inserted the energy due to the contribution of the device whose stiffness is denoted as $\bar{k}_d$ and whose elongation as l_d. Equilibrium requires a constant force in all unfolded domains

$$l_c \frac{k_B T}{l_p} \frac{2-\eta_i}{(1-\eta_i)^2} = \bar{k}_d l_d. \tag{SM34}$$

Moreover, we must take into account the constraint

$$\sum_i \chi_i l_c \eta_i + l_d = d. \tag{SM35}$$

Following[1] the minimization with respect to the χ_i as the total displacement is increased, shows that the links unfold one at a time, when the elastic energy gain equals the unfolding energy Q. The resulting numerical solution is reported in Figure 10 of the main paper and shows the possibility of describing the curvature effect of the equilibrium branch. For the numerical calculations we have considered an unfolding force $Q = 280\,k_B T$ as found in Eq. SM16.

References

1. De Tommasi, D. *et al.* An energetic model for macromolecules unfolding in stretching experiments, *J. R. Soc. Interface* **10**, 20130651 (2013).
2. Linkea, W.A. *et al.* PEVK domain of titin: an entropic spring with actin-binding properties. *J. Struct. Biol.* **137**, 194–205 (2002).
